# Do We Need to Rethink the Epidemiology and Healthcare Utilization of Parkinson's Disease in Germany?

**DOI:** 10.3389/fneur.2018.00500

**Published:** 2018-06-29

**Authors:** Sebastian Heinzel, Daniela Berg, Sebastian Binder, Georg Ebersbach, Lennart Hickstein, Heinz Herbst, Michael Lorrain, Ingmar Wellach, Walter Maetzler, Gudula Petersen, Niklas Schmedt, Jens Volkmann, Dirk Woitalla, Volker Amelung

**Affiliations:** ^1^Department of Neurology, Christian-Albrechts-University, Kiel, Germany; ^2^Department of Neurodegeneration, Hertie Institute for Clinical Brain Research, University of Tuebingen, Tuebingen, Germany; ^3^inav – Institute for Applied Health Services Research GmbH, Berlin, Germany; ^4^Movement Disorders Clinic, Beelitz, Germany; ^5^InGef – Institute for Applied Health Research Berlin GmbH, Berlin, Germany; ^6^Department of General Practice, Institute for Community Medicine, University Medicine Greifswald, Greifswald, Germany; ^7^Office for Neurology, Stuttgart, Germany; ^8^Nervenarztpraxis Gerresheim, Düsseldorf, Germany; ^9^Office for Neurology/Ev. Amalie Sieveking Hospital, Hamburg, Germany; ^10^Grünenthal GmbH, Aachen, Germany; ^11^Department of Neurology, University Hospital of Würzburg, University of Würzburg, Würzburg, Germany; ^12^Department of Neurology, Sankt Josef Hospital, Bochum, Germany

**Keywords:** Parkinson's disease, epidemiology, insurance claims, mortality, incidence, prevalence, comorbidity, healthcare

## Abstract

Epidemiological aspects of Parkinson's disease (PD), co-occurring diseases and medical healthcare utilization of PD patients are still largely elusive. Based on claims data of 3.7 million statutory insurance members in Germany in 2015 the prevalence and incidence of PD was determined. PD cases had at least one main hospital discharge diagnosis of PD, or one physician diagnosis confirmed by a subsequent or independent diagnosis or by PD medication in 2015. Prevalence of (co-)occurring diseases, mortality, and healthcare measures in PD cases and matched controls were compared. In 2015, 21,714 prevalent PD cases (standardized prevalence: 511.4/100,000 persons) and 3,541 incident PD cases (standardized incidence: 84.1/100,000 persons) were identified. Prevalence of several (co-)occurring diseases/complications, e.g., dementia (PD/controls: 39/13%), depression (45/22%), bladder dysfunction (46/22%), and diabetes (35/31%), as well as mortality (10.7/5.8%) differed between PD cases and controls. The annual healthcare utilization was increased in PD cases compared to controls, e.g., regarding mean ± SD physician contacts (15.2 ± 7.6/12.2 ± 7.3), hospitalizations (1.3 ± 1.8/0.7 ± 1.4), drug prescriptions (overall: 37.7 ± 24.2/21.7 ± 19.6; anti-PD medication: 7.4 ± 7.4/0.1 ± 0.7), assistive/therapeutic devices (47/30%), and therapeutic remedies (57/16%). The standardized prevalence and incidence of PD in Germany as well as mortality in PD may be substantially higher than reported previously. While frequently diagnosed with co-occurring diseases/complications, such as dementia, depression, bladder dysfunction and diabetes, the degree of healthcare utilization shows large variability between PD patients. These findings encourage a rethinking of the epidemiology and healthcare utilization in PD, at least in Germany. Longitudinal studies of insurance claims data should further investigate the individual and epidemiological progression and healthcare demands in PD.

## Introduction

Parkinson's disease (PD) is a common, chronic and progressive neurodegenerative disease often leading to disability, care dependency, reduced quality of life and premature death (1). Moreover, PD is a complex and heterogeneous disease regarding disease etiologies, presentation of symptoms and disease progression (2, 3). In addition to cardinal motor symptoms several non-motor symptoms and comorbidities, such as dementia, depression, and autonomous dysfunction, often affect patients with PD (4–6). Consequently, PD with its associated diseases and complications, and their individual progression over time pose specific, and often complex and multi-faceted management demands. The personal needs of PD patients require professional and patient-centered medical treatment and healthcare support (7).

Despite its relevance there is paucity of recent and real-world estimates of the epidemiology of PD, medical treatment practice and other aspects of healthcare utilization and support of PD patients in Germany. In this context, insurance claims could serve as data basis for gaining insight into the recent epidemiological status of PD including associated diseases and complications as well as the real-world utilization of PD treatments and healthcare services. Thus far, such estimates are often difficult to compare between studies and/or nations as data sources, i.e., primary and secondary data, and diagnostic and inclusion criteria differ (8, 9) and may change over time (10). Consequently, the status of the epidemiology of PD and PD healthcare in Germany and across Europe remains elusive. Both of these aspects should be investigated within one large, recent and national dataset while applying previously used PD case identification criteria, and while investigating PD as a heterogeneous disease with frequently co-occurring/co-morbid diseases/complications.

In Germany epidemiological estimates may be outdated and restricted to elderly individuals [data from 2006, individuals aged 65+ years (11); data from 2004/2007, aged 50+ years (12)]. “Official” reports (7) of prevalence estimates of PD in Germany still refer to estimates by the European Brain Council in 2010 that were however interpolated from prevalences reported for Spain, France, Italy, and UK (13). In addition to the nation-wide prevalence and incidence of PD, health claims data may provide the real-world healthcare utilization of PD patients as important for informed decision-making in healthcare policy and planning.

The present study of the MoPED consortium (Morbus Parkinson Epidemiologie in Deutschland) aimed to (1) provide standardized estimates of the PD prevalence and incidence in Germany in 2015, (2) investigate the prevalence of (co-)occurring diseases/complications and mortality in patients with and without PD, and (3) quantify the real-world PD treatment and healthcare utilization in Germany.

## Methods

### Data source

This study was based on the InGef research database, which contains anonymized patient-level claims data from approximately 6.7 million insured members of several German statutory health insurances. In brief, the database includes demographic information, ambulatory services and diagnoses, hospital data including diagnoses and procedures, reimbursed remedies and aids as well as dispensations of reimbursed drugs. External validity of this database against German population data has been shown previously (14). To create a representative sample regarding the age and sex distribution in Germany and to increase the generalizability of findings, a sample of ~4 million insured persons (4.5% of German population) was drawn from the entire InGef database. For 3,695,024 of those insured persons complete data of the observational period between 2013 and 2015 (including death in 2015) was available and served as study population.

The study protocol and the results of this study were reviewed and discussed with a group of German PD experts composed of hospital neurologists and neurological practices, as well as two German patient organizations (Supplementary Material, online only). Due to the anonymized nature of the data, an informed consent of the study participants and a vote of an external ethic committee were not required.

### Study design and study population

The present study was designed as a retrospective cohort study to estimate the population-based prevalence and incidence of PD per 100,000 persons in Germany in 2015. Moreover, we compared the prevalence of (co-)occurring symptoms and diseases, mortality, and utilization of healthcare resources between prevalent PD cases and individuals without PD.

Subjects were eligible to enter the cohort if they fulfilled the following inclusion criteria: (1) continuous insurance in 2015 or until death, (2) a diagnosis of PD (ICD-10 G20 code) in 2015 (see berrlow), (3) continuous insurance or birth in 2013 and 2014 (baseline period), and (4) absence of a diagnosis of PD in the baseline period (only for calculation for the incidence).

**Figure 1:**
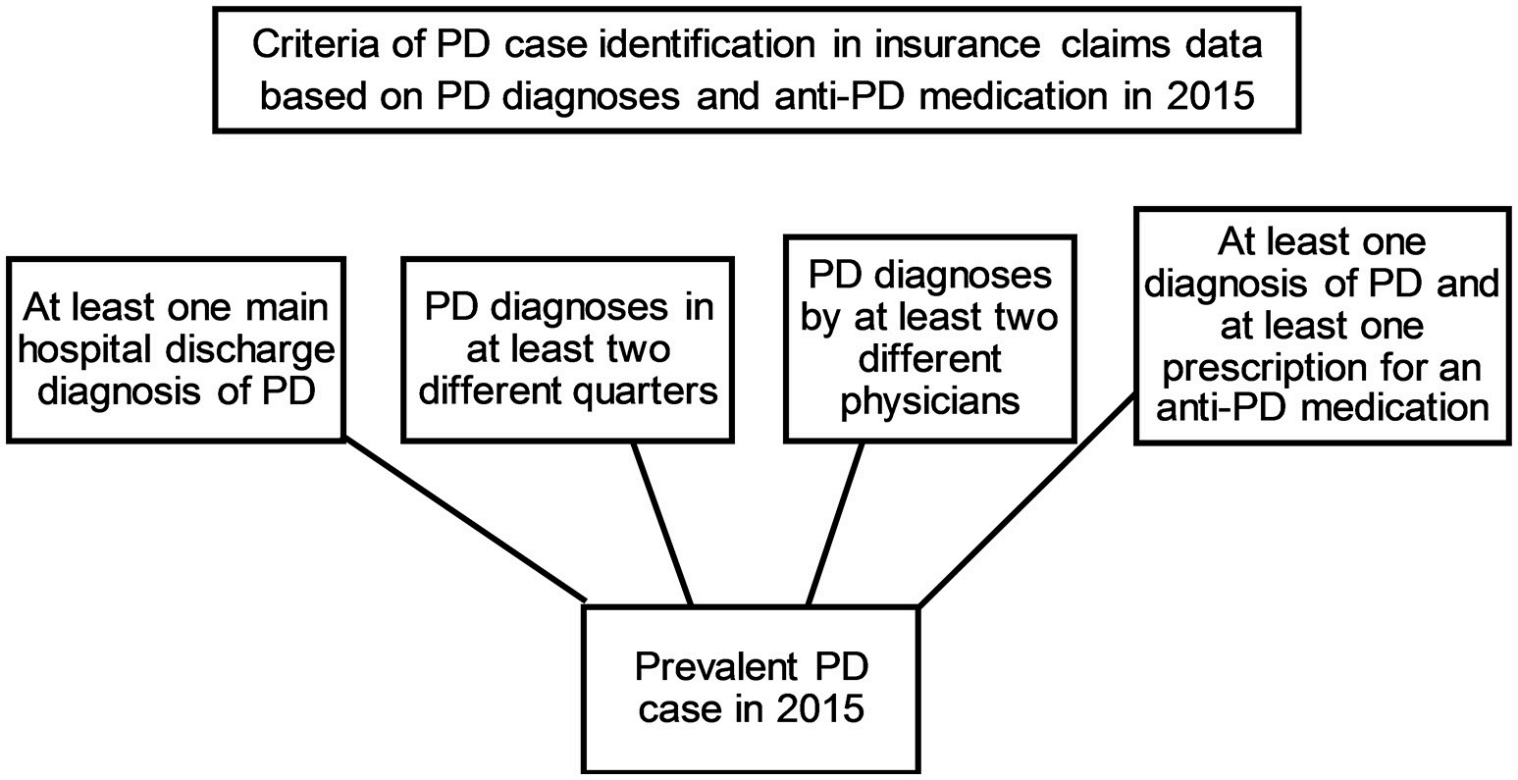
Flow-chart of PD case identification criteria. Of the four different criteria at least one had to be fulfilled in prevalent PD cases in 2015.

Prevalent cases of PD fulfilled one of the following criteria in 2015: (1) Main hospital discharge diagnosis of PD (which can be considered as the main medical reason for hospitalization), (2) at least two diagnoses of PD in two different quarters, (3) PD diagnoses by at least two different physicians, and/or (4) at least one diagnosis of PD and at least one prescription for an antiparkinsonian (anti-PD) medication (ATC codes N04Bx) in 2015 (see flow-chart in Figure 1). Only primary and secondary hospital diagnoses and verified ambulatory diagnoses were considered. As suggested previously (11, 12), we aimed to increase validity of ambulatory diagnoses by at least one subsequent PD diagnosis and by an additional anti-PD drug prescription in 2015. Supporting the validity of the PD diagnosis 79% of prevalent PD cases and 59% of incident PD cases fulfilled more than one of these criteria (Supplementary Material, online only).

To compare diseases/complications and mortality, as well as medications, other treatments and health services between PD patients and insured persons without PD, an age- and sex-matched control group (1:1 matching) without a PD diagnosis in 2015 was selected.

### Definitions of (co-)occurring diseases/complications, mortality, treatments, and healthcare services

Pre-defined (co-)occurring complications/symptoms and diseases frequent in the elderly and/or in PD patients (where their co-occurrence may partly be considered a co-morbidity) were identified based on primary or secondary hospital diagnosis or verified ambulatory diagnosis as defined by ICD-10 codes. The prevalence of diabetes, dementia, depression, hypertension, cancer, sleeping disorders, fatigue, bladder dysfunction and, sexual dysfunction are reported. Patients who deceased in 2015 were identified based on death as reason for disenrollment from the insurance.

Prescriptions of any medication and of different medications (i.e., compounds) were identified based on ATC codes. Anti-PD medications were identified based on “N04Bx” ATC codes while 7-digit ATC codes quantified the number of different of anti-PD medications used in 2015. Moreover, for each prescription the specialty of the prescribing physician was indicated. Furthermore, health claims of therapeutic remedies and aids were specified and quantified. Definitions and codes of all variables are provided in the Supplementary Material (online only).

### Statistical analysis

Prevalence and incidence of PD per 100,000 persons were calculated stratified by sex and age groups (0–17, 18–29, 30–39, 40–49, 50–59, 60–64, 65–69, 70–74, 75–79, 80–84, 85–89, and 90+ years), by dividing the absolute number of PD cases by the number of cohort subjects (or the respective stratum) in 2015. For the prevalence and incidence of PD, 95% confidence intervals were calculated assuming a binominal distribution. In addition, the overall prevalence and incidence were standardized according to the age, sex, and regional distribution (i.e., federal states) of the total German population in 2015 (82.2 million). Differences between controls and PD cases were statistically tested using Mann–Whitney U tests for continuous variables and Chi-square tests for categorical variables. Statistical analyses were performed using SAS Enterprise Guide, version 4.3.

## Results

### Prevalence and incidence of PD

**Figure 2:**
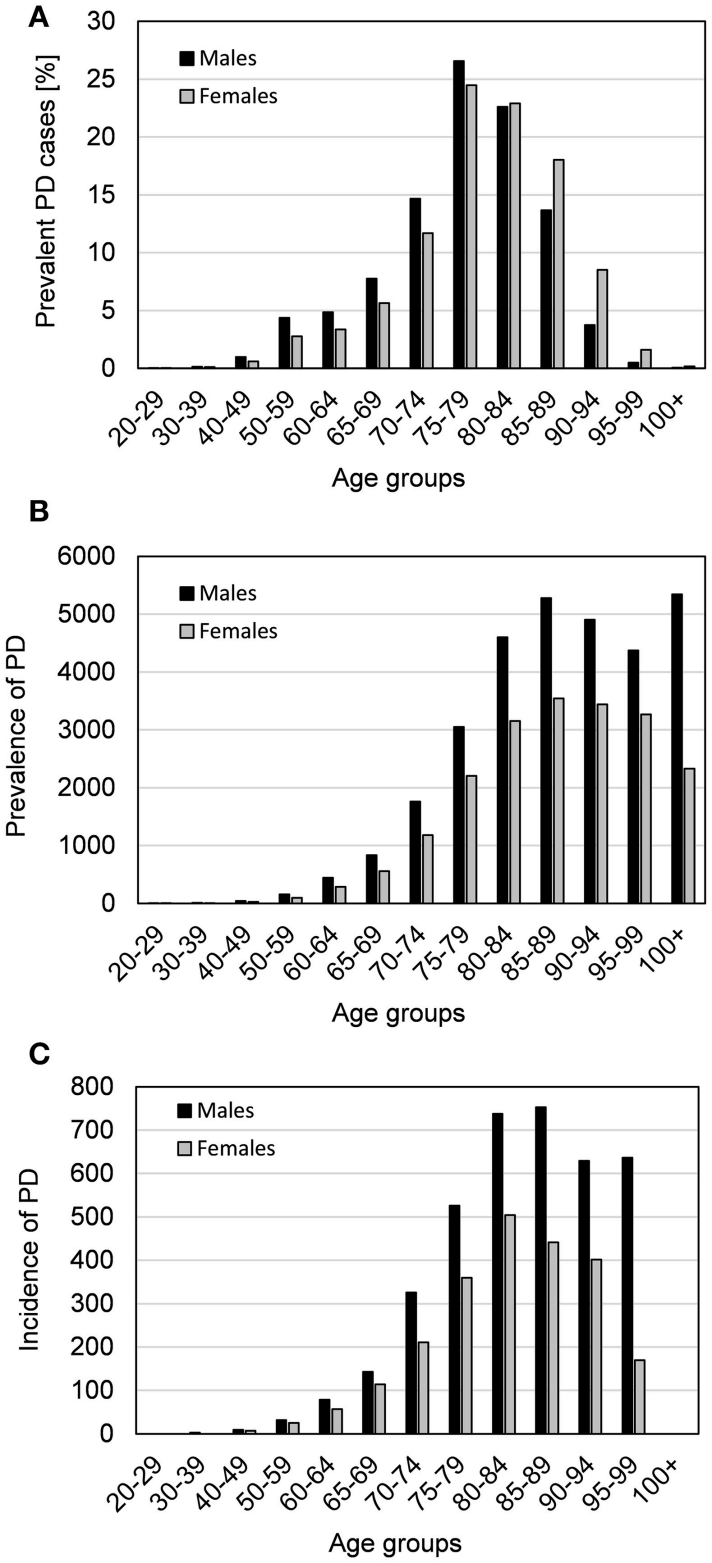
The prevalence and incidence of Parkinson's disease in Germany in 2015. Stratified by age groups and sex **(A)** the percentages of prevalent Parkinson's disease (PD) cases (within sex groups percentages add up to 100%), **(B)** the crude prevalence of PD per 100,000 persons, and **(C)** the crude incidence of PD per 100,000 persons in Germany in 2015 are shown.

Overall, 21,714 prevalent PD cases were identified. PD cases were slightly more frequently male (50.8%) than female (49.2%) and had a mean age (± standard deviation) of 77.8 ± 9.3 years. Males (76.6 ± 9.2 years) were slightly younger than females (79.0 ± 9.1). The crude prevalence of PD in 2015 was 587.7 per 100,000 persons (95%-confidence interval: 579.8–595.5) and the standardized prevalence per 100,000 persons was 511.4 (504.6–518.2). The prevalence and incidence of PD stratified by age and sex are shown in Figures 2A,B and the Supplementary Tables 1, 2 (online only). Males showed a higher prevalence of PD than females at all ages. The statistical projection to general population indicates an overall prevalence of 420.371 PD cases in Germany.

**Figure 3:**
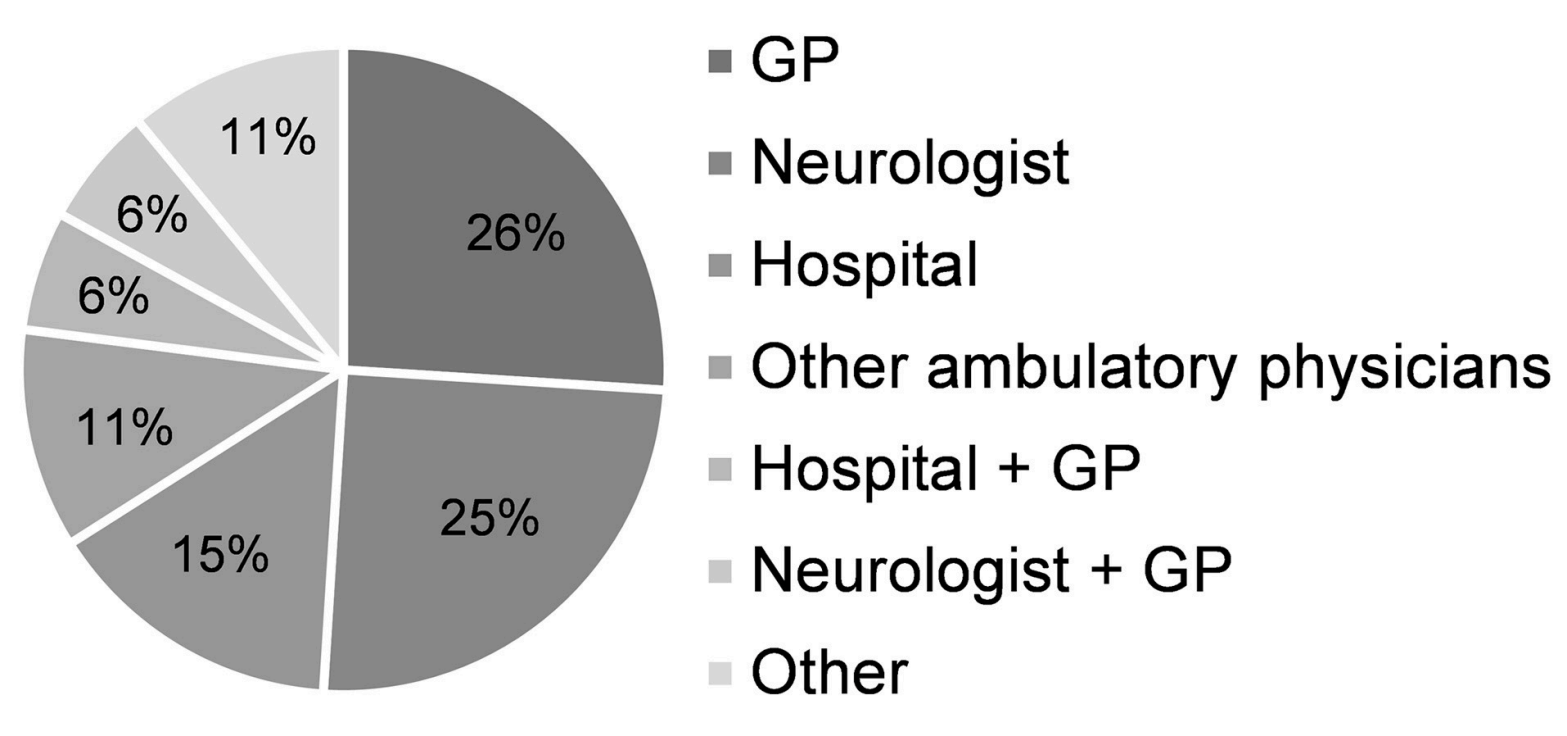
The first diagnosis of PD. Type of physician or medical institution (in percent) giving an initial diagnosis of Parkinson's disease in incident cases.

The crude incidence of PD per 100,000 persons in Germany in 2015 stratified by age and sex is shown in Figure 2C. In 2015, 3,541 incident PD cases were identified corresponding to an overall crude incidence of 95.8 per 100,000 persons (92.7–99.0) and a standardized incidence of 84.1 (95%-confidence interval: 81.3–86.9). Incident PD cases were slightly more frequently male (52.2%) than female (47.8%). The mean age (± standard deviation) was 76.4 ± 9.7 years while males (75.7 ± 9.5) were younger than females (77.1 ± 9.9). The statistical projection to general population of Germany indicates an overall incidence of 69,130 PD cases in Germany in 2015. The first diagnosis of PD in incident cases was made by medical professionals of a variety of institutions, i.e., resident physicians [including neurologists, general practitioners (GP)], ambulatory physicians, hospitals/clinics, or other institutions/professionals. The relative frequencies of these medical institutions and professionals determining the first PD diagnosis are shown in Figure 3.

### (Co-)occurring diseases, complications, and mortality in PD

In particular, dementia, depression, bladder dysfunction, fatigue and sleeping disorders showed a markedly higher prevalence in PD patients compared to controls. PD patients showed also higher prevalence (PD>controls) for diabetes and hypertension. Sexual dysfunction differed only marginally between cohorts, whereas for cancer no difference was observed (Table 1). Moreover, an annual mortality of 10.7% in PD patients (*N* = 2,330 deceased in 2015) was observed, which was significantly (*p* < 0.001) higher than for persons in the control group (*N* = 1.264 deceased; 5.8%). The mortality ratio was 1.84 in PD patients relative to controls.

**Table 1:** (Co-)occurring diseases and complications.

**Diagnosis**	**PD**	**Controls**	***P*-value**
Bladder dysfunction	9.937 (46%)	4.796 (22%)	<0.001
Cancer	4.672 (22%)	4.792 (22%)	>0.1
Dementia	8.506 (39%)	2.845 (13%)	<0.001
Depression	9.807 (45%)	4.766 (22%)	<0.001
Diabetes	7.679 (35%)	6.782 (31%)	<0.001
Fatigue	2.186 (10%)	1.317 (6%)	<0.001
Hypertension	16.951 (78%)	16.501 (76%)	<0.001
Sexual dysfunction	838 (4%)	738 (3%)	0.003
Sleeping disorders	4.414 (20%)	2.670 (12%)	<0.001

*Values indicate the number (percentage) of persons with (associated) diseases/complications*.

### Prescription of medications

Generally, number and diversity of prescribed medications differed between prevalent PD cases and controls (Table 2). PD cases had a 42 and 31% higher number of overall and different number of prescriptions, respectively, than controls. The majority of PD patients (78%) were prescribed anti-PD drugs, yet 22% of PD patients did not receive any prescription of anti-PD drugs. Between PD patients a large variability regarding the total number of anti-PD prescriptions and the number of different anti-PD drugs was observed.

**Table 2:** Prescribed drugs for patients with Parkinson's disease and controls.

**Drug prescription status**	**PD patients**	**Controls**
Persons with any drug prescription	100%	94%
Total prescriptions [mean number per year (SD)]	37.7 (24.2)	21.7 (19.6)
Number of different drugs [mean number per year (SD)]	10.8 (5.6)	7.4 (5.3)
Persons with prescription of anti-PD drugs	78%	2%
Total anti-PD drug prescriptions [mean number per year (SD)]	7.4 (7.4)	0.1 (0.7)
**Persons by number of differing anti-PD drug prescriptions**		
0 anti-PD medications 1 2 3 4 5+	22% 42% 21% 9% 3% 1%	98% 2% 0% 0% 0% 0%

In 66% of PD patients anti-PD drug prescription were at least once made by a neurologist in 2015, whereas in 34% of PD patients no neurologist was involved in the prescription of anti-PD drug prescriptions (Table 3).

**Table 3:** Prescription of anti-PD medication by physician groups in 2015.

**Physician groups prescribing anti-PD medication**	**PD patients**
Patients with anti-PD drug prescription	16.842 (100%)
Only by neurologist	6.686 (40%)
Only by GP	3.951 (23%)
GP and neurologist	3.731 (22%)
Specialist of other medical disciplines	711 (4%)
GP and specialist of other medical disciplines	560 (3%)
Neurologist and specialist of other medical disciplines	746 (4%)
Only specialist of other medical disciplines	457 (3%)

*GP, General practitioner; PD, Parkinson's disease*.

### Treatments and healthcare utilization

Compared to controls, PD patients were treated by a higher number of physicians (20%, 1.2 more physicians), and had a higher overall number of physician contacts (25%, 3.0 more physician contacts) in 2015. The numbers of GP and psychiatrist contacts were similar between PD patients and controls, whereas treatment by a neurologist occurred more than twice a year in PD patients and only 0.3 times per year in controls. Hospital treatment in general was more frequent in PD patients compared to controls. Here, nearly two-fold higher numbers of hospitalizations and total days of hospital treatment in PD patients compared to controls were observed (Table 4). The utilization of other forms of treatment and healthcare services is shown in Table 5. Treatments and support as indicated by therapeutic remedies and aids were provided more frequently for PD patients (in about half of all PD patients) compared to controls (15–30%). More than one third of PD patients was treated with physical therapy, whereas speech therapy, occupational therapy and psychotherapy were relatively rarely prescribed. Complex treatment regimes, such as apomorphine/pump treatment, deep brain stimulation (each <0.1%), and diagnostic polysomnography (2%) were rarely (newly) prescribed in PD patients.

**Table 4:** Physician contacts and hospital treatment in 2015.

**Type of medical healthcare**	**PD patients**	**Controls**
Number of different physicians	7.3 (3.8)	6.1 (3.7)
Total physician contacts	15.2 (7.6)	12.2 (7.3)
GP contacts	5.0 (2.5)	4.5 (2.5)
Neurologist contacts	2.2 (1.9)	0.3 (0.9)
Psychiatrist contacts	0.2 (0.9)	0.1 (0.5)
Any (inpatient) hospital treatment	49%	30%
Hospitalizations	1.0 (1.4)	0.5 (1.1)
Hospital days	9.8 (19.4)	4.8 (14.1)

*Mean (standard deviation) or percent of total are indicated*.

**Table 5:** Other treatments, diagnostic measures and healthcare services in PD patients and controls.

**Healthcare claims**	**PD patients**	**Controls**
Aids	47%	30%
Any therapeutic remedy	58%	28%
Physical therapy	36%	2%
Speech therapy	4%	<0.4%
Occupational therapy	6%	1%
Psychotherapy	1%	<0.1%
Apomorphine treatment	<0.1%	<0.1%
Pump treatment	<0.1%	<0.1%
Deep brain stimulation	<0.4%	<0.1%
Complex treatment	2%	<0.1%
Polysomnography	2%	1%
Residential care	21%	6%

## Discussion

This study provides recent epidemiological estimates of standardized prevalence and incidence of PD based on representative statutory insurance claims data in Germany in 2015. Moreover, the analysis provides the recent prevalence of associated diseases and complications, as well as up-to-date quantifications of the healthcare utilization of PD patients and a control population.

### Prevalence and incidence of PD

Results of studies reporting crude and standardized prevalence and incidence of PD differ widely (8, 15). While global and regional differences in the epidemiology of PD may exist (8), inconsistencies between studies may often be due to methodological differences inherent to data sources and definitions of PD case ascertainment that vary in sensitivity and specificity.

In Germany, a standardized prevalence of 797 and 961/100,000 PD cases (year 2004 and 2007), and a standardized incidence of 192–229/100,000 person-years (2004–2010) based on statutory insurance claims data of the “Allgemeine Ortskrankenkasse” (AOK) and criteria of repeated PD diagnosis (in- and outpatient) and PD diagnosis confirmed by anti-PD medication have been reported (12). In comparison, the standardized prevalence of 511/100,000 and standardized incidence of 84/100,000 persons as shown in the present study are markedly lower. These inconsistencies might be best explained by methodological differences. Unlike the InGef research database (14) used in the present study, the AOK database only included persons with 50+ years of age and overall reported higher mortality rates than the age-stratified general German population (12). Also, regional and potential socioeconomic differences between samples might underlie the higher PD prevalence and incidence rates in the AOK data.

Insurance claims data in France from 2010 showed, using specific and sensitive PD criteria, respectively, a substantially lower standardized PD prevalence and incidence compared with our findings, i.e., 308–410 PD cases/100,000 persons and annual incidence rates of 36–49 PD cases/100,000 persons. In Italy, a standardized prevalence of 233 PD cases/100,000 persons and standardized incidence rate of 23.1 PD cases/100,000 persons based on primary care data from 2013 have been shown (16). Thus, large differences in epidemiological estimates of PD exist between studies and countries. The rather conservative PD definition (11) and the external validity of the data basis (14) suggest a realistic representation of the epidemiology of PD in Germany in 2015 with a projected prevalence of 420,371 PD patients and a projected incidence of 69,130. However, several limitations inherent to insurance claims data have to be considered (see below). The present estimates are substantially higher than the projected prevalence of 219,579 PD patients in Germany referred to by the European Brain Council in 2010 (13) that however was projected from prevalence estimates reported for France, Italy, Spain, and UK. This outdated estimate, that is not specific for Germany, is still “officially” reported (7).

In about one quarter of incident PD cases the first diagnoses was made solely by residential neurologists and one quarter by residential general practitioners, whereas about one half was first diagnosed by other institutions (i.e., hospitals or ambulatory physicians) and other medical professionals. Thus, similar to previous findings (12) a large percentage of PD patients received the initial diagnosis by professionals other than neurologists. Previously, confirmation rates of PD diagnoses based on post-mortem histopathology of 74% for non-experts, and 80–84% for medical specialists have been shown (17). While misdiagnoses of PD may lead to an overestimation of the prevalence and incidence of PD, the number of PD cases who remain undiagnosed is also unknown. The extent to which these aspects contribute to an over- or underestimation of the prevalence and incidence of PD has to be further investigated, e.g., based on long-term longitudinally confirmed PD diagnoses, and age of PD onset and PD severity data that may indicate delayed PD diagnoses.

### (Co-)occurring diseases/complications, and mortality in PD

PD often presents as a more complex and heterogeneous clinical phenotype than defined through its cardinal motor symptoms alone. Accordingly, a high prevalence of several PD-associated diseases and complications was observed. Numerous other studies (4–6) already showed non-motor diseases/complications to be frequent in PD and for some of these the present study showed an even higher prevalence. Among the preselected diseases/complications, dementia was diagnosed in 39% of PD patients and 13% of controls, which was higher than 25–30% prevalence of dementia in PD patients reported previously (18, 19). Depression was diagnosed in 45% of PD patients and 22% of controls, whereas German secondary insurance claims data previously showed a prevalence of depression in 33% of PD patients (11). While in meta-analysis a similar overall prevalence of depression in PD was observed the study also showed large heterogeneity in findings and methodological differences suggesting that prevalence estimates based on different studies might not be exact (20). Bladder dysfunction was also substantially more frequent in PD patients (46%) than controls (22%). Similarly, another study reported about a two-fold relative risk of bladder symptoms in PD patients compared to controls (21), confirming bladder dysfunction as a major complication in PD. Sleeping disorders were observed in 20% of PD patients and 12% of controls. This prevalence in PD is low compared to previous reports (22), but unlike specific clinical studies sleeping disorders may not be assessed or coded in many medical practices. Thus, claims data may not represent the actual prevalence of sleeping disorders in PD. A similar explanation may apply for fatigue (10% of PD patients), which differed in prevalence from previous findings [about one third of PD patients (23)]. Hypertension was very frequent in PD (78%) as well as controls (76%). Possibly more specific aspects, e.g., orthostatic hypotension, might show larger differences between PD and controls. Consistent with a meta-analysis of associations between PD and diabetes (24) PD patients (35%) were more often diabetics than controls (31%). Cancer prevalences did not significantly differ between PD patients and controls. However, cancer types were not differentiated which should be considered since melanoma may increase, whereas other types of cancer may decrease the PD risk (25).

Importantly, PD-associated diseases, such as depression, dementia and autonomous dysfunction, may exert a larger impact on quality of life in many PD patients than the characteristic PD motor symptoms (26).

In the present study, the mortality rate in PD patients was almost twice as high as in controls. A meta-analysis showed standardized mortality ratios ranging from 0.9 to 3.8 (pooled mortality ratio: 1.4) (27), thus the mortality ratio of 1.84 in the present study was within the mid-range of previous reports.

### Medical healthcare utilization

The number of different prescriptions was generally high but realistic for an elderly population in Germany. PD patients were prescribed a relatively low mean number of 1.3 different anti-PD drugs with however large differences between patients. PD patients (22%) often did not receive any anti-PD drugs, although the treatment guidelines recommend pharmacological treatment in early-stage PD (7). Some of these PD patients might still be under observation (before a definite PD diagnosis) or might have secondary Parkinsonism, e.g., vascular Parkinsonism, without anti-PD medication prescriptions. A vast number of PD patients (42%) was treated with a monotherapy, which might be adequate for some PD patients (e.g., *de-novo* PD, advanced age), while some may benefit from a more differentiated anti-PD medication plan. However, the exact L-dopa equivalent daily dose (LEDD) could not be calculated from the insurance claims data, yet longitudinal changes in prescribed medication (with increasing disease severity) constitute an interesting outcome of longitudinal studies.

The prescription of anti-PD drugs in 2015 was in only 40% of PD patients exclusively made by neurologists, and did in 67% of PD patients involve a neurologist at least once (in addition to other medical professionals). However, PD patients would benefit from an annual consultation of a neurologist, since PD specialist may best adjust treatments under consideration of (changing) factors and (non-)motor symptoms (7, 28). In 2015, PD patients only had three physician contacts more than controls. The higher prevalence of comorbidities in PD than controls and PD-specific demands of treatment and specialist consultation suggests this difference to be relatively low. PD patients consulted neurologists on average 2.2 times per year however as indicated by the standard deviation of 1.9 neurologist contacts large heterogeneity was observed between PD patients. Some evidence suggests that neurologist treatment is associated with higher survival rates, lower rates of hip-fractures and lower nursing home placements compared to non-specialist treatment (29). However, the mere number of physician contacts might only provide incomplete measure of the medical services. Hospital admissions were more frequent in PD patients compared to controls (49/30% of individuals), however hospital days showed large variance. Possibly hospital treatment due to accidents might have been prevented in some PD patients if optimal PD treatment and management had been provided (29).

Aids were provided for less than half of PD patients (47%) and 30% of controls. Aids can constitute highly relevant means of support, but the individual demands and insurance coverage of aids might differ between PD patients.

More than 42% of PD patients were not prescribed therapeutic remedies. In particular, only 36% of PD patients had physical therapy, despite growing evidence showing beneficial effects of exercise on various aspects of daily living in PD patients (30). Complex treatments (2%), and (new prescriptions of) infusion pumps and deep brain stimulation (<0.1%) were relatively rare. Residential care was more often required for PD patients (21%) than for controls (6%), but to what extent decreased mobility or (co-)occurring diseases/complications underlie the demand for residential care needs to be further investigated.

### Limitations

Several limitations of the present study have to be considered. (1) The insurance claims data was standardized to the general population regarding age, sex, and region, and thus for these factors the present study can be considered representative. However, other factors including socio-economic status could not be accounted for, which may partly limit the representativeness. (2) The accuracy of PD diagnosis might differ between professionals, and we therefore employed several approaches to decrease the rate of false-positive PD cases, e.g., through repeated/independent PD diagnosis or diagnosis confirmed by anti-PD medication criteria. (3) Reported estimates may partly represent an “administrative” prevalence and incidence as in some cases the PD diagnosis might have been given due to reasons of reimbursement. Thus, insurance claims data may partly be biased toward overestimating epidemiological estimates of PD. (4) As not covered by insurances, additional treatments/services as needed by PD patients might not be identified through insurance claims data.

Claims data constitute a valuable data source for up-to-date longitudinal observational studies in (prodromal) PD research. Based on this data, diagnoses and insurance claims that were made (many) years after (or before) the PD diagnosis can be analyzed regarding temporal sequences of diagnoses, medication and healthcare utilization in PD and/or in groups defining possible PD subtypes. Moreover, PD can be defined as a syndrome and not one disease entity (31), and may be embedded within disease networks with overlapping disease characteristics. Due to the comprehensive availability of diagnoses, treatments, and healthcare utilization across all medical disciplines, insurance claims are a promising data source to address contemporary and complex research questions in medical sciences.

We believe the present study encourages a rethinking of the epidemiology and healthcare utilization in PD: (1) Compared to previous reports, substantially higher prevalence and incidence of PD in Germany in 2015 were observed in this comprehensive analysis of insurance claims data. Here, PD cases below the age of 50 years, i.e., early onset PD, were also considered and a both sensitive and specific case identification strategy was applied. (2) While largely consistent with previous findings, the co-occurrence of non-motor symptoms and complications was very frequent in PD patients. Yet, while motor as well as non-motor symptoms often pose substantial healthcare demands, large variability between PD patients regarding the number of treatments and medications was observed. Possibly, many PD patients would benefit from additional and holistic healthcare utilization. Basic and clinical PD research continue to require political, societal and industry support. However, the agendas of PD research as well as healthcare policy making should both consider changes in the epidemiology of PD and individual needs of PD patients.

## Conclusion

The projected standardized prevalence of PD in the present study was nearly two-fold higher than suggested by previous “official” estimates. Several associated diseases and complications including dementia, depression, diabetes, and bladder dysfunction known to pose additional burden to PD patients and to require additional healthcare were highly frequent in PD patients. The healthcare utilization differed strongly between PD patients, and demands of substantial and holistic treatment and support for many PD patients are possibly unmet so far. Longitudinal studies of insurance claims data are needed to investigate the individual and epidemiological progression of PD and (holistic) healthcare demands of PD patients.

## Author contributions

SH study design, first draft of manuscript, interpretation of results. DB, SB, DW, WM, NS, HH, GE, ML, JV, and IW study design, interpretation of results, revision of manuscript. GP study organization and design, revision of manuscript. LH study design, statistical analyses, interpretation of results, revision of manuscript. VA study design, revision of manuscript, interpretation of results.

### Conflict of interest statement

DB reports other from UCB Pharma, other from Lundbeck, other from Prexton Therapeutics, from GE-Healthcare, personal fees from UCB Pharma, personal fees from BIAL, personal fees from Bayer, grants from Michael J. Fox Foundation, Janssen Pharmaceutica N.V., German Parkinson's Disease Association (dPV), BMWi, BMBF, Parkinson Fond Deutschland gGmbH, UCB Pharma, TEVA Pharma, EU, Novartis Pharma, and Lundbeck, outside the submitted work. SB reports that the Institute for Applied Health Service Search received financial support from Grünenthal to perform the study. GE reports personal fees from Grünenthal GmbH, outside the submitted work. LH reports grants from inav—privates Institut für angewandte Versorgungsforschung GmbH, during the conduct of the study. HH reports personal fees from Grünenthal GmbH, Abbvie, and Bayer AG outside the submitted work. ML reports personal fees from Grünenthal GmbH, during the conduct of the study; personal fees from Medtronic, AbbVie, Licher, UCB, Merck, Biogen, Servier, Bayer, Teva, Zambon, and Bial, outside the submitted work. IW reports personal fees and non-financial support from Grünenthal GmbH, during the conduct of the study; personal fees and non-financial support from AbbVie GmbH & Co.KG, personal fees and non-financial support from UCB Pharma GmbH, Zambon GmbH, and Bial GmbH, outside the submitted work; and Collaboration with the Deutsche Parkinson Vereinigung (dPV). WM reports grants from FP7 EU, Robert Bosch Foundation, Michael J. Fox Foundation, Neuroalliance, Lundbeck, and Janssen, and personal fees from Abbvie, UCB, GlaxoSmithKline, Licher MT, and Rölke Pharma, outside the submitted work; In addition, WM has a patent for the assessment of dyskinesias licensed to German patent office, 102015220741.2; WM was also invited to Advisory Boards of Market Access & Pricing Strategy GmbH and Abbvie. GP is an employee of Grünenthal GmbH. NS reports grants from inav—privates Institut für angewandte Versorgungsforschung GmbH, during the conduct of the study. JV reports grants and personal fees from Medtronic, personal fees from St. Jude, grants and personal fees from Boston Scientific, personal fees from UCB, Merz, Allergan, TEVA, Novartis, AbbVie, and Grünenthal, outside the submitted work. DW reports support from Grünenthal during the conduct of the study. VA reports that the Institute for Applied Health Service Search received financial support from Grünenthal to perform the study. The remaining author declares that the research was conducted in the absence of any commercial or financial relationships that could be construed as a potential conflict of interest.
