# Supplementary material for: Do We Need to Rethink the Epidemiology and Healthcare Utilization of Parkinson's Disease in Germany?
Source: Front Neurol. 2018 Jun 29;9:500. doi: 10.3389/fneur.2018.00500 (PMC6033992; doi:10.3389/fneur.2018.00500)
Supplement: Supplementary file 1 [file Data_Sheet_1.pdf]

Supplementary material (online only)

1) The MoPED consortium

Jutta Ahmerkamp-Böhme, Volker Amelung, Daniela Berg, Sebastian Binder, Johannes Bonjean, Georg Ebersbach, Sebastian Heinzel, Heinz Herbst, Lennart Hickstein, Michael Lorrain, Walter Maetzler, Friedrich-Wilhelm Mehrhoff, Gudula Petersen, Niklas Schmedt, Jens Volkmann, Ingmar Wellach, Dirk Woitalla, and the patient organizations Jung & Parkinson e.V. and Deutsche Parkinson Vereinigung e.V..

MoPED
Morbus Parkinson
Epidemiologie in Deutschland

2) Fulfillment of PD criteria in prevalent and incident PD cases

Supplementary Figure 1: Fulfillment of multiple PD criteria

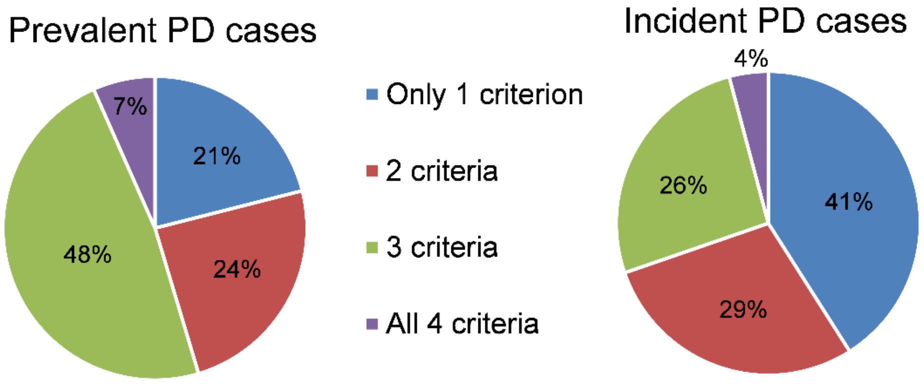

Fulfillment of individual PD criteria in prevalent and incident PD cases

Criterion #1: Primary hospital diagnosis of PD in 2015: 8% prevalent PD cases, 9% incident PD cases

Criterion #2: At least two diagnoses of PD in two different quarters in 2015: 90% prevalent PD cases, 65% incident PD cases

Criterion #3: At least two PD diagnoses by two different physicians in 2015: 64% prevalent PD cases, 38% incident PD cases

Criterion #4: At least one diagnosis of PD in 2015 and at least one prescription for an anti-PD medication in 2015: 78% prevalent PD cases, 81% incident PD cases

3) Supplementary Table 1: The prevalence of PD in Germany in 2015

Sex	Age	n (PD cases)	n (overall cohort)	Crude proportion per 100,000 persons	Lower 95%-CI	Upper 95%-CI
Females and males	0-9	<5	262.129	<5	<5	<5
	10-19	<5	352.897	<5	<5	<5
	20-29	10	377.955	2,7	1,0	4,3
	30-39	27	434.090	6,2	3,9	8,6
	40-49	174	530.161	32,8	27,9	37,7
	50-59	779	610.910	127,5	118,6	136,5
	60-64	895	246.326	363,3	339,5	387,1
	65-69	1.459	211.435	690,1	654,6	725,5
	70-74	2.866	197.324	1.452,4	1.399,3	1.505,6
	75-79	5.548	214.772	2.583,2	2.515,2	2.651,2
	80-84	4.943	131.805	3.750,2	3.645,7	3.854,8
	85-89	3.435	82.928	4.142,2	4.003,6	4.280,7
	90-94	1.322	34.833	3.795,3	3.590,7	3.999,8
	95-99	228	6.555	3.478,3	3.026,8	3.929,8
	100+	25	904	2.765,5	1.681,4	3.849,6
	TOTAL	21.714	3.695.024	587,7	579,8	595,5
Males	0-9	<5	134.477	<5	<5	<5
	10-19	<5	181.176	<5	<5	<5
	20-29	5	191.624	2,6	0,3	4,9
	30-39	16	218.132	7,3	3,7	10,9
	40-49	109	268.049	40,7	33,0	48,3
	50-59	483	307.813	156,9	142,9	170,9
	60-64	535	120.055	445,6	407,9	483,4
	65-69	855	102.762	832,0	776,3	887,8
	70-74	1.618	91.850	1.761,6	1.675,7	1.847,4
	75-79	2.931	96.016	3.052,6	2.942,1	3.163,1
	80-84	2.494	54.208	4.600,8	4.420,2	4.781,4
	85-89	1.507	28.558	5.277,0	5.010,6	5.543,4
	90-94	413	8.423	4.903,2	4.430,4	5.376,1
	95-99	55	1.257	4.375,5	3.219,1	5.531,9
	100+	7	131	5.343,5	1.385,0	9.302,0
	TOTAL	11.028	1.804.531	611,1	599,7	622,5
Females	0-9	<5	127.652	<5	<5	<5
	10-19	<5	171.721	<5	<5	<5
	20-29	5	186.331	2,7	0,3	5,0
	30-39	11	215.958	5,1	2,1	8,1
	40-49	65	262.112	24,8	18,8	30,8
	50-59	296	303.097	97,7	86,5	108,8
	60-64	360	126.271	285,1	255,7	314,6
	65-69	604	108.673	555,8	511,5	600,1
	70-74	1.248	105.474	1.183,2	1.117,6	1.248,9
	75-79	2.617	118.756	2.203,7	2.119,3	2.288,1
	80-84	2.449	77.597	3.156,1	3.031,1	3.281,1
	85-89	1.928	54.370	3.546,1	3.387,8	3.704,4
	90-94	909	26.410	3.441,9	3.218,1	3.665,6
	95-99	173	5.298	3.265,4	2.778,8	3.752,0
	100+	18	773	2.328,6	1.252,8	3.404,3
	TOTAL	10.686	1.890.493	565,3	554,5	576,0

4) **Supplementary Table 2:** The incidence of PD in Germany in 2015

Sex	Age	n (PD cases)	n (overall cohort)	Crude proportion per 100,000 persons	Lower 95%-CI	Upper 95%-CI
Females and males	0-9	<5	262.129	<5	<5	<5
	10-19	<5	352.897	<5	<5	<5
	20-29	<5	377.955	<5	<5	<5
	30-39	7	434.090	1,6	0,4	2,8
	40-49	43	530.161	8,1	5,7	10,5
	50-59	171	610.910	28,0	23,8	32,2
	60-64	166	246.326	67,4	57,1	77,6
	65-69	271	211.435	128,2	112,9	143,4
	70-74	521	197.324	264,0	241,4	286,7
	75-79	932	214.772	434,0	406,1	461,8
	80-84	791	131.805	600,1	558,3	642,0
	85-89	455	82.928	548,7	498,3	599,1
	90-94	159	34.833	456,5	385,5	527,4
	95-99	17	6.555	259,3	136,1	382,6
	100+	<5	904	<5	<5	<5
	TOTAL	3.541	3.695.024	95,8	92,7	99,0
Males	0-9	<5	134.477	<5	<5	<5
	10-19	<5	181.176	<5	<5	<5
	20-29	<5	191.624	<5	<5	<5
	30-39	5	218.132	2,3	0,3	4,3
	40-49	24	268.049	9,0	5,4	12,5
	50-59	96	307.813	31,2	25,0	37,4
	60-64	94	120.055	78,3	62,5	94,1
	65-69	147	102.762	143,1	119,9	166,2
	70-74	299	91.850	325,5	288,6	362,4
	75-79	505	96.016	526,0	480,1	571,8
	80-84	400	54.208	737,9	665,6	810,2
	85-89	215	28.558	752,9	652,2	853,5
	90-94	53	8.423	629,2	459,8	798,6
	95-99	8	1.257	636,4	195,4	1.077,5
	100+	<5	131	<5	<5	<5
	TOTAL	1.850	1.804.531	102,5	97,9	107,2
Females	0-9	<5	127.652	<5	<5	<5
	10-19	<5	171.721	<5	<5	<5
	20-29	<5	186.331	<5	<5	<5
	30-39	<5	215.958	<5	<5	<5
	40-49	19	262.112	7,3	4,0	10,5
	50-59	75	303.097	24,7	19,1	30,3
	60-64	72	126.271	57,0	43,9	70,2
	65-69	124	108.673	114,1	94,0	134,2
	70-74	222	105.474	210,5	182,8	238,2
	75-79	427	118.756	359,6	325,5	393,7
	80-84	391	77.597	503,9	453,9	553,8
	85-89	240	54.370	441,4	385,6	497,3
	90-94	106	26.410	401,4	325,0	477,8
	95-99	9	5.298	169,9	58,9	280,9
	100+	<5 VS	773	<5 VS	<5 VS	<5 VS
	TOTAL	1.691	1.890.493	89,5	85,2	93,7

5) Variable definitions and register/administrative codes

Medical diagnoses were coded according to the German modification of the International Classification of Diseases, 10th revision (ICD-10-GM). Prescribed medications were indicated using codes of the Anatomical Therapeutic Chemical (ATC) classification system.

Non-pharmacological treatments, other forms of medical care (e.g. remedies, operations) and support (e.g. aids) were specified based on OPS codes ("Operationen- und Prozedurenschlüssel"; official German adaptation of the International Classification of Procedures in Medicine, ICPM) and EBM codes ("Einheitlicher Bewertungsmaßstab"; catalogue of charges of German statutory insurances).

The specialty of the prescribing physician (e.g. neurologist, general practitioner) was indicated by FG codes. For a list of the respective codes, see below:

Supplementary Table 3: List of variable names, codes and definitions

Variable	Operational definition
Age	Age at 1st January 2015
Sex	Sex at 1st January 2015
Region	Place of residence at 1st January 2015:<br>• federal state<br>• urban/rural<br>• unknown<br>Patients are assigned to the groups based on the municipality key. The municipality key can be traced back to the region of residence and hence it is possible to determine whether patients live in urban or rural areas. However, for a small proportion of patient, the municipality key is not available. These patients will be considered in the unknown category.

Prescribed rehabilitation services	Patients with EBM code 01611 “Verordnung von medizinischer Rehabilitation“ (yes. no)
Residence in residential care or nursing home	Patients with EBM codes 01415 “Dringender Besuch eines Patienten in beschützenden Wohnheimen bzw. Einrichtungen bzw. Pflege- oder Altenheimen mit Pflegepersonal“, 14314 „Zusatzpauschale kontinuierliche Mitbetreuung eines Patienten mit einer psychiatrischen Erkrankung in beschützenden Einrichtungen oder Pflegeheimen“, 16231 „Zusatzpauschale kontinuierliche Mitbetreuung in beschützenden Einrichtungen oder Heimen“, 21231 „Zusatzpauschale Kontinuierliche Mitbetreuung in beschützenden Einrichtungen oder Heimen“ OR a hospital discharge cause 10 „Entlassung in eine Pflegeeinrichtung“ (yes. no)
Dementia	Patients with a primary or secondary hospital diagnosis or verified ambulatory ICD-10 diagnosis F00x-F03x, F051, G30x, G311
Depression	Patients with a primary or secondary hospital diagnosis or verified ambulatory ICD-10 diagnosis F204, F313-F315, F32x, F33x, F341, F412, F432
Sleeping disorders	Patients with a primary or secondary hospital diagnosis or verified ambulatory ICD-10 diagnosis F51x, G47x
Fatigue	Patients with a primary or secondary hospital diagnosis or verified ambulatory ICD-10 diagnosis F480, R53
Bladder dysfunction	Patients with a primary or secondary hospital diagnosis or verified ambulatory ICD-10 diagnosis N31x, N328, N393, N394, R32, R33, R391
Sexual dysfunction	Patients with a primary or secondary hospital diagnosis or verified ambulatory ICD-10 diagnosis F522, N484
Antiparkinson drug	Patients with at least one prescription with an ATC code N04B
Number of different antiparkinson drugs	Number of different drugs used on a seven digit ATC code level with an ATC code N04B
Drug prescription by GP	Patients with at least one prescription with an ATC code N04B by a GP (FG=02 ‘”Arzt/Praktischer Arzt” OR FG=03 ‘”Internist (Hausarzt)”)
Drug treatment by neurologist	Patients with at least one prescription with an ATC code N04B by a neurologist (FG=51 “Nervenheilkunde” OR FG=53 ‘”Neurologe”)
Levodopa	Patients with at least one prescription with an ATC code N04BA01, N04BA03

Amantadine	Patients with at least one prescription with an ATC code N04BB01
Dopamine agonists	Patients with at least one prescription with an ATC code N04BC
Monoamine oxidase B inhibitors	Patients with at least one prescription with an ATC code N04BD
Other dopaminergic agents	Patients with at least one prescription with an ATC code N04BX
Deep brain stimulation	Patients with at least one prescription with an OPS-code 50282, 50283, 50285, 50286, 50289, 5028a-c in the hospital setting
Hospitalizations	Number of hospitalizations with at least one day between the discharge date of a previous hospitalization
Hospitalized person time	Number of days in hospital
Sick leave days	Number of sick leave days
Specialty of the PD diagnosing physician<br>(only for incident cases)	Specialty of the physician diagnosing PD in incident cases (categorical)<br>• Hospital<br>• GP (FG=01 “Allgemeinmediziner” OR FG=02 ‘”Arzt/Praktischer Arzt” OR FG=03 ‘”Internist (Hausarzt)”<br>• Neurologist (FG=51 “Nervenheilkunde” OR FG=53 ‘”Neurologe”)<br>• Other
Ambulatory physician contacts	Number of ambulatory physician visits per quarter per physician
General practitioner contacts	Number of ambulatory visits per quarter per physician with FG=01 “Allgemeinmediziner” OR FG=02 ‘”Arzt/Praktischer Arzt” OR FG=03 ‘”Internist (Hausarzt)”
Neurologist contacts	Number of ambulatory visits per quarter per physician with FG=51 “Nervenheilkunde” OR FG=53 ‘”Neurologe”
Psychiatrist/psychotherapist contacts	Number of ambulatory visits per quarter per physician with FG=58 “Psychiatrie und Psychotherapie” OR FG=61 “Psychotherapeutisch tätiger Arzt” OR FG=68 “Psychologischer Psychotherapeut”
Otolaryngologist contacts	Number of ambulatory visits per quarter per physician with FG=19 “Hals-Nasen-Ohren Heilkunde”

Orthopaedist contacts	Number of ambulatory visits per quarter per physician with FG=10 “Orthopäde”
Internist contacts	Number of ambulatory visits per quarter per physician with FG=23 “Internist”
Different drugs	Number of different prescribed drugs on the seven digit ATC code level
Anticholinergic drugs	Patients with at least one prescription with an ATC code N04Ax
Opioids	Patients with at least one prescription with an ATC code N02Ax
Non-steroidal anti-inflammatory drugs	Patients with at least one prescription with an ATC code M01Ax
Anti-dementia drugs	Patients with at least one prescription with an ATC code N06Dx
Antidepressants	Patients with at least one prescription with an ATC code N06Ax
Antipsychotics	Patients with at least one prescription with an ATC code N05Bx
Occupational therapy indicated for PD	Patients with at least one prescription of remedies with HM_Indikationsschluessel = “EN2” with in €<br>Source: http://www.heilmittelkatalog.de/files/hmk/ergo/en2.htm
Physical therapy indicated for PD	Patients with at least one prescription of remedies with HM_Indikationsschluessel = “ZN2” with in €<br>Source: http://www.heilmittelkatalog.de/files/hmk/physio/zn2.htm
Speech therapy indicated for PD	Patients with at least one prescription of remedies with HM_Indikationsschluessel = “SC1 OR SP6” in €<br>Source: http://www.heilmittelkatalog.de/files/hmk/ergo/SC1.htm<br>http://www.heilmittelkatalog.de/files/hmk/ergo/SP6.htm
Psychotherapy	Patients with at least one prescription of remedies with an EBM-Code 35200-35225 or an OPS-Code 941x
Complex treatment	Patients with at least one ambulatory or hospital OPS code 897dx
Patients with apomorphine treatment	Patients with at least one ambulatory or hospital OPS code 897e0, 897e1
Patients with other drug pump treatment	Patients with at least one ambulatory or hospital OPS code 897e2, 897e3
